# Supplementary material for: Complexome profiling of the Chlamydomonas psb28 mutant reveals TEF5 as an early PSII assembly factor
Source: Plant Cell. 2025 Mar 17;37(6):koaf055. doi: 10.1093/plcell/koaf055 (PMC12164590; doi:10.1093/plcell/koaf055)
Supplement: koaf055_Supplementary_Data [file koaf055_supplementary_data.zip › psb28_interactive.zip!/plots/intensity/qualityPlots/peptides/CYN26-2_Cre03.g174750.t1.2.html]

### Cre03.g174750.t1.2

Trivial name: CYN26-2  
SeqCoverage [median]: 13.70  
Mass: 33.07 kDa  
  
 

| Sequence | MissedCleavage | Mass [Da] | wtIntensity | muIntensity | Score |
| --- | --- | --- | --- | --- | --- |
| FASLAAGR | 0 | 791.43 | 1654.24 | 839.15 | 105.39 |
| NDNPYFQAGK | 0 | 1152.52 | 719.74 | 405.18 | 124.23 |
| VVEGGELLER | 0 | 1099.59 | 2071.02 | 1981.16 | 127.40 |
|  |  |  | 4445.0 | 3225.5 |  |
